# Potential roles of inorganic phosphate on the progression of initially bound glucopyranose toward the nonenzymatic glycation of human hemoglobin: mechanistic diversity and impacts on site selectivity

**DOI:** 10.1080/23312025.2018.1425196

**Published:** 2018-01-10

**Authors:** Brandy A. Smith, Christina R. Mottishaw, Andria J. Hendricks, Jason Mitchell, Stephanie Becker, Pamela S. Ropski, Bomina Park, Marie Finkbeiner-Caufield, Barbara Garay-Nontol, R.W. Holman, Kenneth J. Rodnick

**Affiliations:** 1 Department of Chemistry, Idaho State University Pocatello, Idaho 83209; 2 Department of Biological Sciences, Idaho State University Pocatello, Idaho 83209

**Keywords:** glycated hemoglobin, glucose, phosphate, protein binding, water

## Abstract

Nonenzymatic glycation (NEG) begins with the non-covalent binding of a glucopyranose to a protein. The bound glucopyranose must then undergo structural modification to generate a bound electrophile that can reversibly form a Schiff base, which can then lead to Amadori intermediates, and ultimately to glycated proteins. Inorganic phosphate (Pi) is known to accelerate the glycation of human hemoglobin (HbA), although the specific mechanism(s) of Pi as an effector reagent have not been determined. The aim of this study was to determine whether Pi and a glucopyranose can concomitantly bind to HbA and react while bound within the early, noncovalent stages to generate electrophilic species capable of progress in NEG. ^31^P and ^1^HNMR of model reactions confirm that bimolecular reactions between Pi and glucopyranose occur generating modified glucose electrophiles. Computations of protein/substrate interactions predict that Pi can concomitantly bind with a glucopyranose in HbA pockets with geometries suitable for multiple acid/base mechanisms that can generate any of four transient electrophiles. Pi-facilitated mechanisms in the noncovalent stages predict that the glycation of β-Val1 of HbA to HbA1c is a “hot spot” because the β-Val1 pocket facilitates many more mechanisms than any other site. The mechanistic diversity of the Pi effect within the early noncovalent stages of NEG predicts well the overall site selectivity observed from the *in vivo* glycation of HbA in the presence of Pi. These insights extend our basic understanding of the NEG process and may have clinical implications for diabetes mellitus and even normal aging.

## Introduction

Glucose is a vital substrate for energy metabolism of all cells, yielding ATP under aerobic and anaerobic conditions. Glucose also reacts nonenzymatically with intracellular and extracellular proteins to form covalently-modified proteins (also referred to as glycated proteins), both *in vivo* (Bunn, Haney, Gabbay & Gallop, 1975; Bunn, Haney, Kamin, Gabbay & Gallop, 1976; Stevens, Vlassara, Abati & Cerami, 1977) and *in vitro* (Mohammad, Fraenkel-Conrat & Olcott, 1949; Lea & Hannan, 1949; Bookchin & Gallop, 1968) over prolonged periods of time. This process, termed nonenzymatic glycation (NEG), is a significant feature of diabetes mellitus, in which persistent hyperglycemia leads to the accumulation of advanced glycation endproducts (AGE). NEG is associated with pathophysiological outcomes, as well as age-related chronic diseases such as microangiopathy, retinopathy, and nephropathy (Brownlee, 1995).

The most studied NEG reactions are those between glucose and adult human hemoglobin (HbA) yielding HbA1c. HbA1c has a modified glucose bound to the N-terminal valine of one or both of the HbA β-chains and is increased in diabetic patients (Little & Sacks, 2009). Measurement of HbA1c has been useful as a clinical marker of long-term (6–8 weeks) glycemic control and is an assessment of the risk of developing cardiovascular disease and diabetic complications.

**Figure 1.**
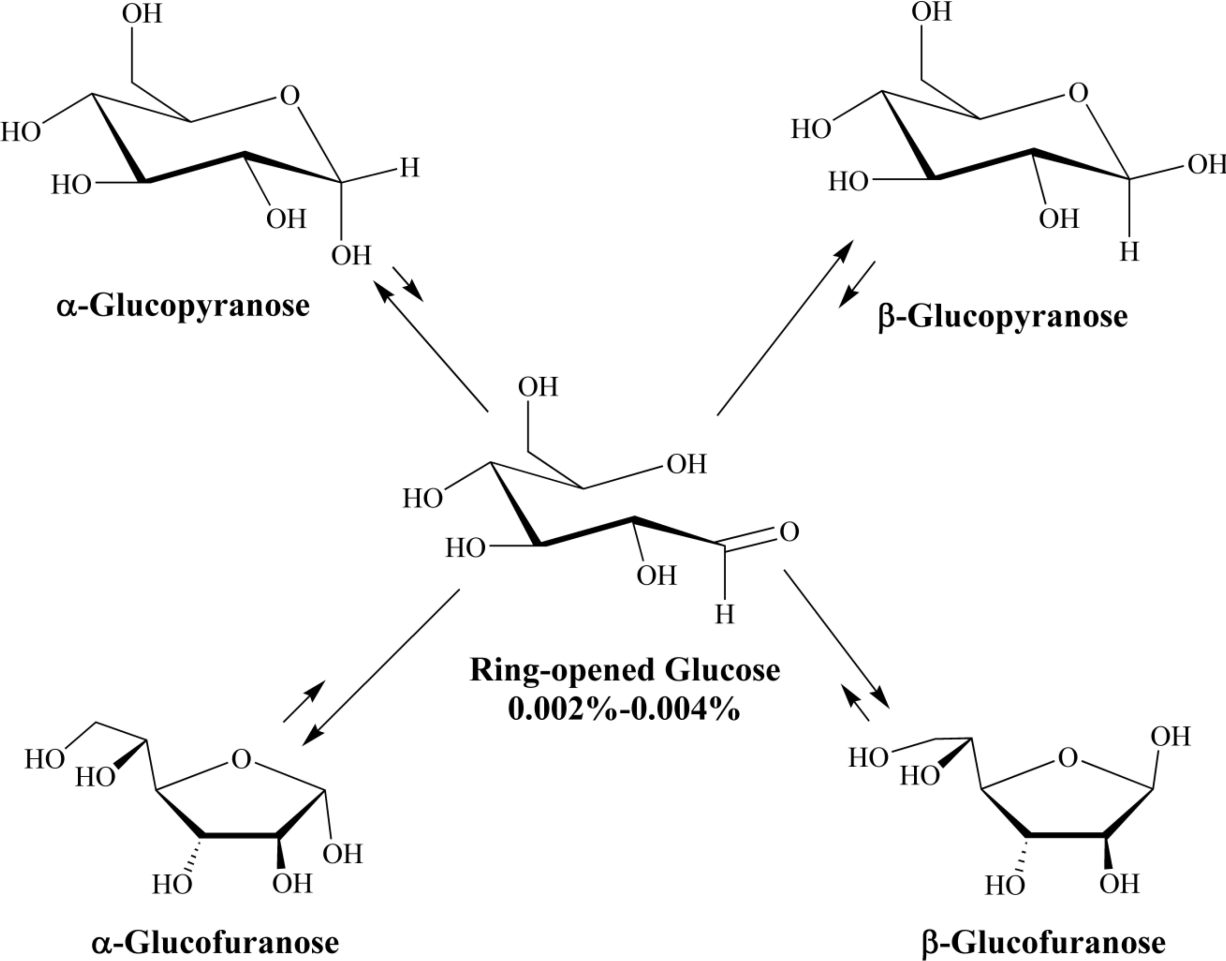
The five interconverting isomers of glucose observed in aqueous media. The four ring-closed isomers interconvert through a transient, central ring-opened isomer (2). The relative length of equilibrium arrows generally reflects the extent of reversibility. The ring-closed glucopyranoses (34% α, 65% β) and the glucofuranoses (ca. 1% together) constitute ca. 49,999 of 50,000 glucose isomers in dynamic equilibrium. The lifetime of the ring-opened isomer is less than the NMR time scale at room temperature and thus exists in the ring-open form for less than 10^−4^ seconds (Bryant, 1983). Further, the transient ring-opened isomer can generate a hydrate that is not shown. As extrapolated to NEG chemistry, the ring-opened isomer is sufficiently electrophilic to react with R-NH_2_ amino acid residues inside a protein pocket. The two pyranoses (α and β, collectively referred to in the text as 1) and the two furanoses (α and β) are not sufficiently electrophilic and are not predicted to react with amino acid residues. The ring-closed glucose isomers bind with greater affinity to HbA than does the ring-opened isomer (Clark et al., 2013). In view of this binding differential, the low concentration and the short lifetime, it is asserted that the ring-opened isomer is not the species that initially binds to HbA (Clark et al., 2013; Wang et al., 2013; Park et al., 2016; Rodnick et al., 2017).

Our research goal was to elucidate the potential roles of inorganic phosphate on the progression of initially bound glucopyranose toward the nonenzymatic glycation of human hemoglobin, with a focus on mechanistic diversity and impacts on site selectivity. More specifically, to better understand what reagents and mechanisms are involved that enable a noncovalently bound glucose isomer to proceed in NEG. Five interconverting glucose isomers exist in reversible equilibrium in the erythrocyte cytosol, including a pair of ring-closed glucopyranoses (α and β; at 34% and 65% respectively) and a pair of ringclosed glucofuranoses (α and β combined at ca. 1%, Figure 1). The ring-closed species are not sufficiently electrophilic (able to accept two electrons in the formation of a new bond) to significantly react with protein amino acid residues and progress in NEG. The fifth structure, through which the four ring-closed isomers interconvert, is a ring-opened isomer containing an aldehyde group. This species *is* sufficiently electrophilic to react with amino acid residues and progress in NEG. However, the direct binding of the reactive ring-opened isomer of glucose in NEG is unlikely. The equilibrium concentration of the ringopened glucose in aqueous solution is 0.002% (Bunn & Higgins, 1981) or less, corresponding to just 0.12 μM in erythrocyte cytosol. This is likely an overestimate of the reactive concentration of available ringopened isomer since reversion to a ring-closed isomer occurs rapidly, less than the NMR timescale of 10^4^ seconds (Bryant, 1983), making it a transient electrophile. It is therefore anticipated that reversion to ring-closed isomers occurs at a rate faster than ring-open isomer binding. In contrast, the ring-closed isomers of glucose are at 50,000 times the concentration, are long-lived, and bind better to HbA than does the ring-opened isomer (Clark, Santin, Bryant, Holman & Rodnick, 2013). Moreover, the initially bound glucose species to human serum albumin (HSA) have been experimentally determined to be ringclosed isomers (Wang et al., 2013). This observation was confirmed by Clark et al. (2013) for HbA and is consistent with studies of glucose and other monosaccharides binding to several enzymes (Lee, Bagdasarian, Meng & Zeikus, 1990; Allen et al., 1994; Thoden et al., 2003; Lee & Jeffrey, 2005).

**Figure 2.**
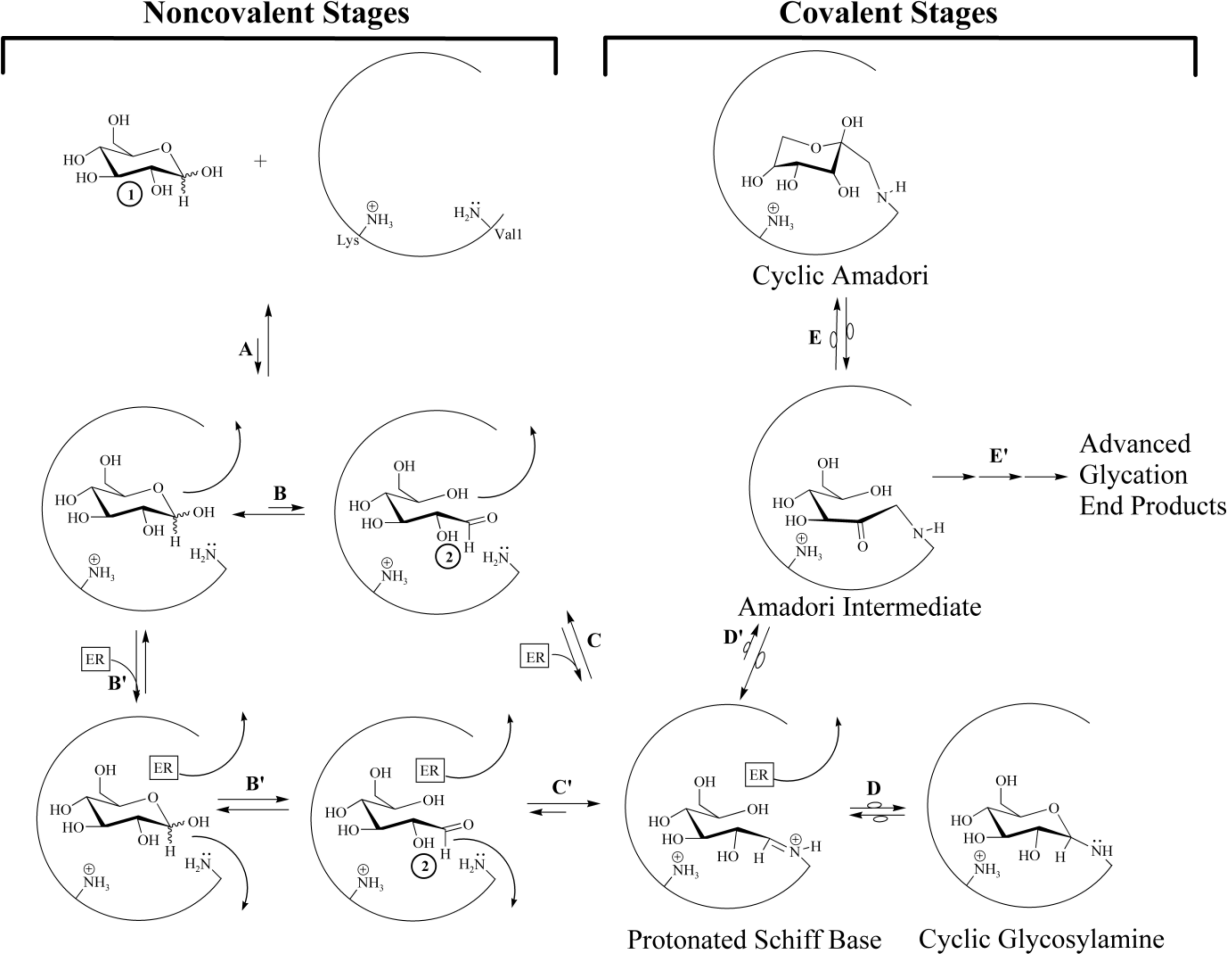
The NEG process in the formation of HbA1c involving transient intermediate 2, including routes possible with or without the participation of an effector reagent. A glucopyranose (1) is exposed to a protein pocket (an internal pocket or at the surface of the protein) and undergoes reversible, noncovalent binding (process A). 1 may dissociate from the protein pocket or it may proceed via B or B’. Following path B, under the influence of amino acid residues only, acid/base reactions with 1 generate a noncovalently bound, reactive transient electrophile 2. Intermediate 2 may dissociate from the protein pocket, reverse back to 1, or it may proceed via C to the protonated Schiff base (via reaction with the Val1 R-NH_2_ serving as the nucleophile). The covalently bound, protonated Schiff base has three fates: it can reverse back to 2 , it can isomerize to a cyclic glycosylamine via D, or it can undergo Amadori formation via D’. The Amadori intermediate has three fates; it can reverse back to the protonated Schiff base (D’), it can isomerize to a cyclic Amadori (E), or it can proceed via multiple mechanisms to AGE (E’). If concommitant binding of a Pi (mono or dianionic) with 1 occurs (Path B’), the Pi can function as an effector reagent (ER) and undergo acid/base reactions to generate 2. The concommitantly bound Pi with 2 has multiple fates: Pi can dissociate, 2 can dissociate, or 2 can undergo reaction with the Val1 R-NH_2_ serving as the nucleophile and generate the protonated Schiff base (path C’). The protonated Schiff base then has the same fate as above, proceeding through D or D’. Amadori from this path has the same fates as above. Note: Amadori formation may or may not involve an ER. Note 2: arrows with loops in them represent intramolecular isomerizations.

In order to proceed in NEG, the initially-bound glucopyranose must undergo modification, while bound, to become sufficiently electrophilic before exiting the protein pocket (for the general scheme of NEG, see Figure 2).

**Figure 3.**
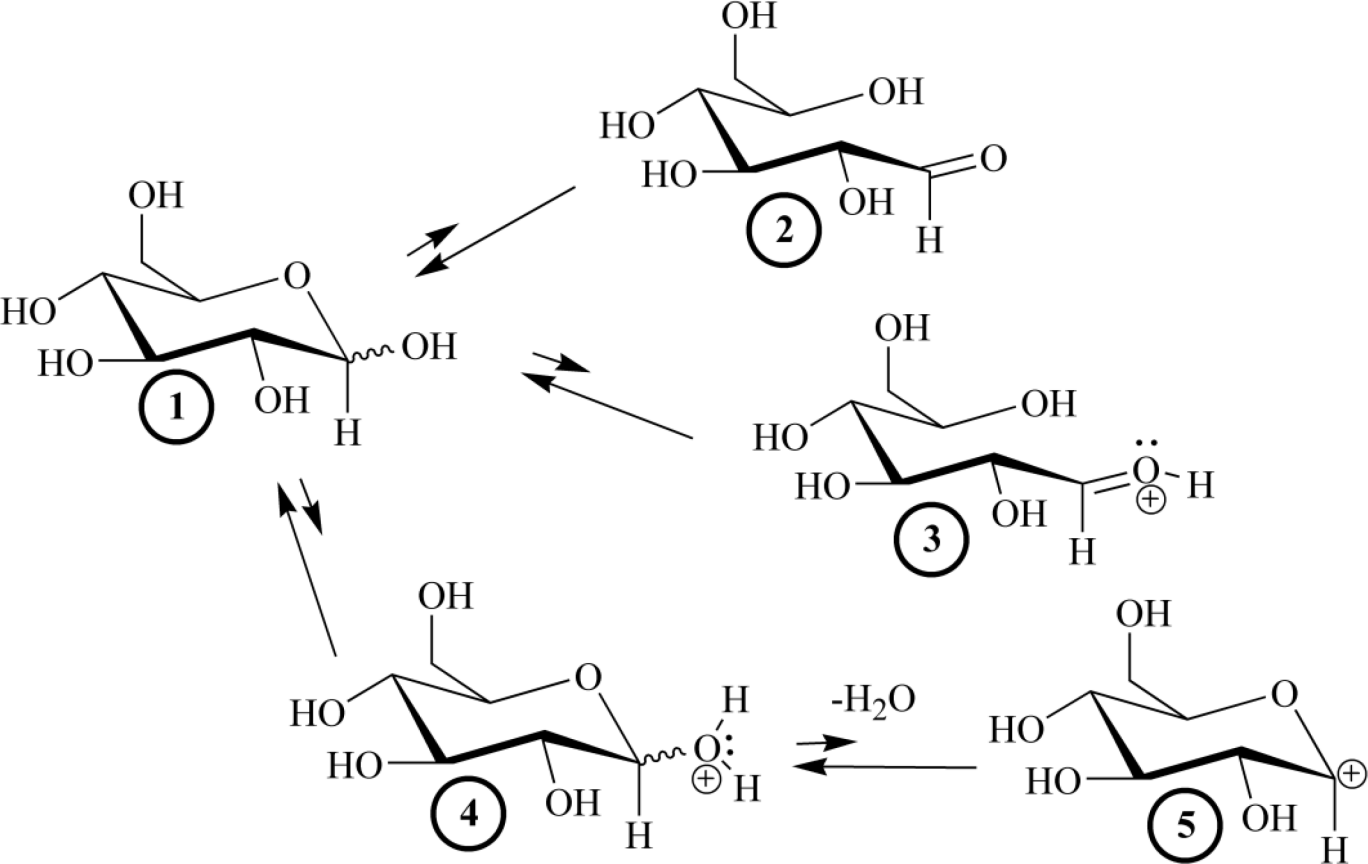
The four modified, transient electrophiles (2–5) that can theoretically be generated from a glucopyranose, 1. Structure 1 represents both the α- and β-glucopyranose isomers. Structures 2–5 are the transient, electrophilic species that can theoretically be generated from a glucopyranose, 1. The relative lengths of equilibrium arrows indicate that structures 2–5 are short-lived, unstable species that rapidly convert back to 1. 2 is generated from 1 via protonation of the hemiacetal oxygen and the concerted deprotonation of the anomeric OH. 3 can be generated from 1 through the protonation of the hemiacetal oxygen followed by ring-opening. If protonation at the anomeric OH occurs, 4 is generated which can then lose water as a leaving group to yield 5. Species 2–5 are the most probable electrophiles involved in NEG.

Four potential modified, transient electrophilic glucose species can be reversibly generated from a bound glucopyranose (Structures 2–5, Figure 3). Irrespective of the specific structure or formation process, the electrophile can then be attacked by a nucleophilic amino acid residue such as N-terminal valines on the β-chains of HbA and proceed through NEG. For an amino acid residue to act as a nucleophile (donate two electrons in the formation of a new bond with the electrophilic modified glucose) it must be in an R-NH_2_ amine form.

Several investigators report that inorganic phosphate (Pi) increases the rate of NEG for certain proteins (Watkins, Neglia-Fisher, Dyer, Thorpe & Baynes, 1987; Gil, Mata-Segreda & Schowen, 1988; Kunika, Itakura & Yamashita, 1989; Gil, Peña, Vásquez & Uzcategui, 2002), although not all proteins (Watkins et al., 1987). For HbA, a mechanism(s) for accelerating glycation has not been fully established. That said, most of the mechanisms that have been proposed place the Pi effect (here defined as glycation facilitation by Pi) at Amadori formation or later (Watkins et al., 1987; Gil et al., 1988; Kunika et al., 1989; Gil et al., 2002; Gil, Vasquez, Peña & Uzcategui, 2004; Gil, Salcedo & Romero, 2005; Ito, Nakahari & Yamamoto, 2011). Recently, Rodnick, Holman, Ropski, Huang & Swislocki (2017) posited that Pi plays multiple mechanistic roles within the pre-Amadori, early noncovalent stages of HbA glycation. Specifically, Pi is asserted to facilitate the modification of the initially-bound glucopyranose to more reactive species.

The first goal of this work was to assess how Pi serves to modify the initially-bound glucopyranose to generate a bound transient electrophile. In addition, what transient electrophilic species can theoretically be formed through modification of a bound glucopyranose? Is there mechanistic diversity involving Pi, and if so, how does it impact site selectivity in HbA glycation? Site selectivity was defined here as the relative extent of glycation between HbA pockets and within a given HbA pocket. A clinically-relevant objective was to better understand why Val1 of β-chains (HbA1c) predominates over other potential HbA glycation sites and to understand why HbA1c formation occurs so slowly (over weeks to months).

## Results

### ^1^HNMR assessment for the Pi-facilitated formation of reactive electrophiles from a glucopyranose in aqueous media.

**Figure 4.**
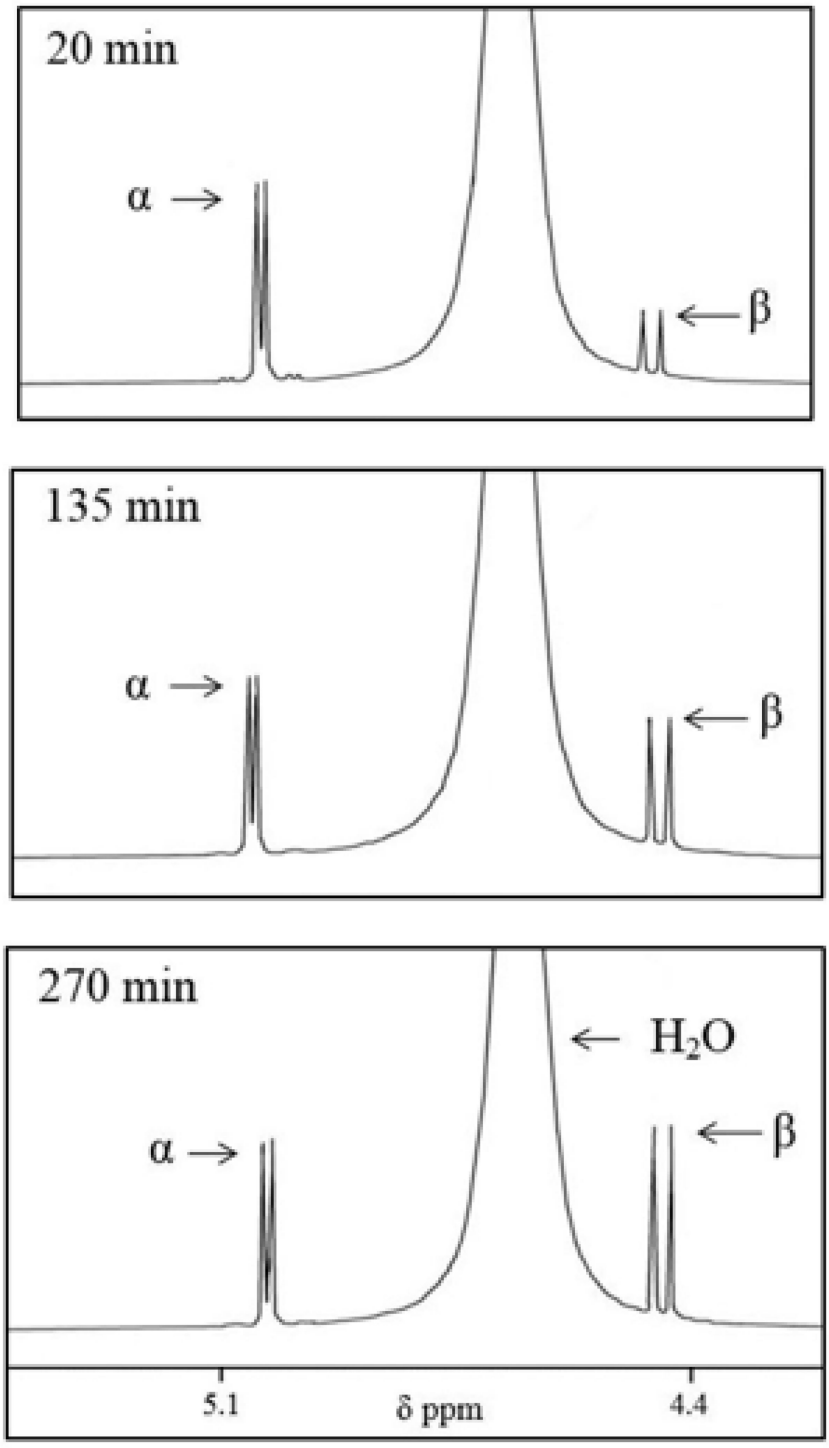
^1^HNMR of pure α-glucopyranose in D_2_O at 20°C followed as a function of time. Three representative spectra are shown as the α-glucopyranose isomer converted to the β-glucopyranose isomer. The initial time point was measured ca. 8 minutes and followed until the time for inflection was reached. The doublet at 5.06 ppm is assigned to the proton on the anomeric carbon of the α-glucopyranose. The doublet at 4.47 ppm is assigned to the proton on the anomeric carbon of the β-glucopyranose. The large, broad singlet at 4.66 ppm is water contamination in the D_2_O solvent. The bottom spectrum is the 270 min point designated as the inflection time where α- and β-glucopyranose are found in equal concentration.

First, pure α-glucopyranose was placed in aqueous solution (D_2_O) and its reversible interconversion with the β-glucopyranose isomer was followed as a function of time by ^1^HNMR at room temperature (Table 1, Experiment 1). The extent of α-glucopyranose to β-glucopyranose interconversion through the ring-opened glucose isomer (Figure 1) was measured and expressed in terms of the time required for inflection to occur. Inflection is here defined as the time required for the formation of a 50:50 mixture of α- and β-glucopyranose isomers from pure α-glucopyranose (Figure 4, Table 1).

The inflection time in the presence of equimolar dianionic Pi (98% dianionic and 2% monoanionic at pH = 9.1) in D_2_O is 8 min, an approximate 30-fold decrease relative α- to β-glucopyranose inflection in D_2_O alone (270 min) (Table 1, Experiment 2). The inflection time observed with monoanionic Pi (4% dianionic and 96% monoanionic at pH = 4.5) is 215 min, reflecting an enhancement of α- to β-glucopyranose by a factor of 1.25 (Table 1, Experiment 3). Inflection times for α-G6P to β-G6P are shorter than can be measured (less than 5 min) (Table 1, Experiment 4).

### ^1^HNMR validation that Pi-facilitated formation of reactive electrophiles is the result of Pi structural effect and not a pH effect.

The inflection times for six model potential effector reagents (some physiological – histidine, taurine, lactate, bicarbonate, and 2,3-BPG – and one non-physiological – metformin), whose pH values range from 4.2 to 9.1 (Experiments 5–10) are shown in Table 1. The reagents were evaluated for mechanistic purposes to discern whether inflection times are governed by effector reagent structure or whether there is a significant pH effect. We observed a large variation in inflection times and inflection times did not trend with pH. Experiments 11–14 were conducted to further assess the facilitation of electrophile formation from α-glucopyranose as affected by Pi mixtures in the physiological pH range (6.4–7.4). Over the physiological pH range Pi clearly facilitates the formation of electrophiles.

### ^31^PNMR and computational validation of a structural effect of Pi: The formation of a glucopyranose:Pi adduct:

The ^31^P chemical shift for Na_2_HPO_4_ in D_2_O (in the absence of glucopyranose) was observed to be 2.78 ppm, while that for NaH_2_PO_4_ was 0.47 ppm. When Na_2_HPO_4_ was placed in a 1:1 molar ratio with α-glucopyranose, a single broadened phosphorous signal at 1.77 ppm was observed. At a 1:5 ratio, the single broadened signal was at 1.66 ppm, while at a 5:1 ratio the signal, again broadened, was at 2.13 ppm. If the Pi were not interacting and making a reversible complex with glucose, the ^31^P chemical shift should be at 2.78 ppm. That the broadened, single peak observed in a 1:1 dibasic Pi to glucopyranose was at 1.77 ppm in a solution at pH 9.1 makes certain that a new phosphorus environment exists, consistent with a reversible adduct between dibasic Pi and glucopyranose. As computational validation (ab initio HF631G**), energies were calculated for separated α-glucopyranose and Pi and compared to a geometry optimized adduct between α-glucopyranose and Pi. The formation of the bridging adduct was 30 kcal/mol exothermic relative to isolated starting materials. Together, the ^31^PNMR and ab initio calculations suggest that the primary structural effect of Pi is to bridge with a glucopyranose, enabling the generation of transient electrophiles.

### Computational assessment of Pi concomitant binding with glucopyranose isomers in HbA.

Binding of dianionic Pi with α-glucopyranose is predicted to take place in three of the four HbA pockets known to be glycated on each β-chain (Table 2). These pockets include the β-Val1pocket (containing Val1/Lys8/82/132/144), the Lys17/120 pocket, and the Lys59/61/65/66 pocket. Dianionic Pi is also predicted to concomitantly bind with β–glucopyranose in the β-Val1 pocket. Both α- and β– glucopyranose bind with monoanionic Pi in all four HbA β–chain pockets (including the Lys95 pocket). These concomitant binding events occur with exothermicities comparable with both glucopyranose binding alone (−5.0 to −2.9 kcal/mol) and Pi binding alone [−3.6 to −3.2 kcal/mol, Table 2).

### Computational assessment of geometrically feasible mechanisms for the Pi-assisted formation of 2–5 from a glucopyranose within the β-Val1 pocket of HbA.

**Figure 5.**
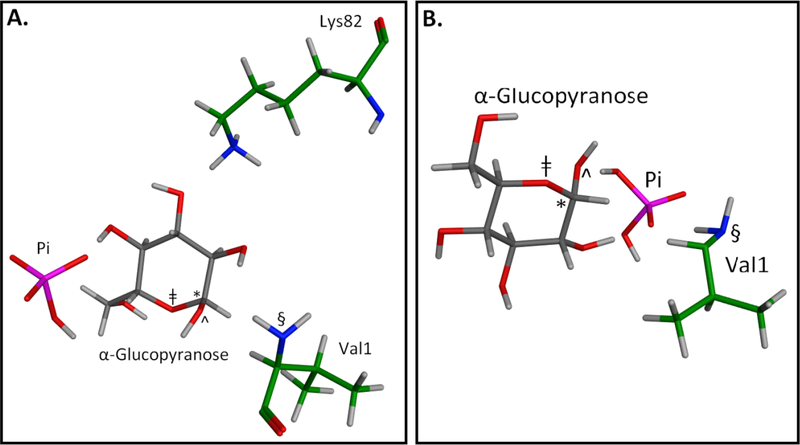
Two MOE geometries representative of energetic minima whereby Pi and a glucopyranose concomitantly bind in the β-Val1 pocket of HbA. For visual clarity, only those residues needed to illustrate the geometrically viable mechanisms are shown.</P/>* anomeric carbon; ^ anomeric OH; ǂ hemiacetal oxygen; § nucleophile. Red is oxygen; blue is nitrogen; pink is phosphorous; gray are glucopyranose carbons; green are amino acid residue carbons; white are hydrogens.</P/>A. From this geometry, three distinct mechanisms are possible. In one mechanism the dianionic Pi bridges the glucopyranose, protonates the hemiacetal oxygen (dist. = 4.11Å) and deprotonates the anomeric OH (dist. = 4.23Å) as Val1 attacks the incipient anomeric carbonyl in a concerted fashion (dist = 5.13Å) on route to a Schiff base (equating to Figure 5, mechanism I). In a second mechanism, the Lys82 protonates dianionic Pi (dist. = 1.14Å) as the Pi protonates the anomeric OH (dist. = 4.11Å), making the anomeric carbon electrophilic and generating water as a leaving group as the Val 1 attacks as a nucleophile in a concerted fashion (dist. = 5.13Å) on route to a cyclic glycosylamine (equating to Figure 6, Mechanism VIa). In a third mechanism, the Lys82 protonates dianionic Pi (dist. = 1.14Å) as the Pi protonates the hemiacetal oxygen of the glucopyranose (dist. = 4.11Å), making the anomeric carbon electrophilic as the Val 1 attacks as a nucleophile in a concerted fashion (dist. = 5.13Å), generating 3 (equating to Figure 6, Mechanism IV) on route to a protonated Schiff base. </P/>B. From this geometry, three distinct mechanisms are possible. In one mechanism the monoanionic Pi bridges the glucopyranose, protonates the hemiacetal oxygen (dist =4.19Å) and deprotonates the anomeric OH (dist. = 3.60Å) as Val1 attacks the incipient anomeric carbonyl in a concerted fashion (dist. = 4.91Å) on route to a Schiff base (equating to Figure 5, mechanism I). In a second mechanism, the monoanionic Pi protonates the anomeric OH (dist. = 1.87Å), making the anomeric carbon electrophilic and generating water as a leaving group as the Val 1 attacks as a nucleophile in a concerted fashion (dist. = 4.91Å) on route to a cyclic glycosylamine (equating to Figure 6, Mechanism VIa). In a third mechanism, monoanionic Pi protonates the hemiacetal oxygen of the glucopyranose (dist. = 4.19Å), making the anomeric carbon electrophilic as the Val 1 attacks as a nucleophile in a concerted fashion (dist. = 4.91Å), generating 3 (equating to Figure 6, Mechanism IV) on route to a Schiff base.

**Figure 6.**
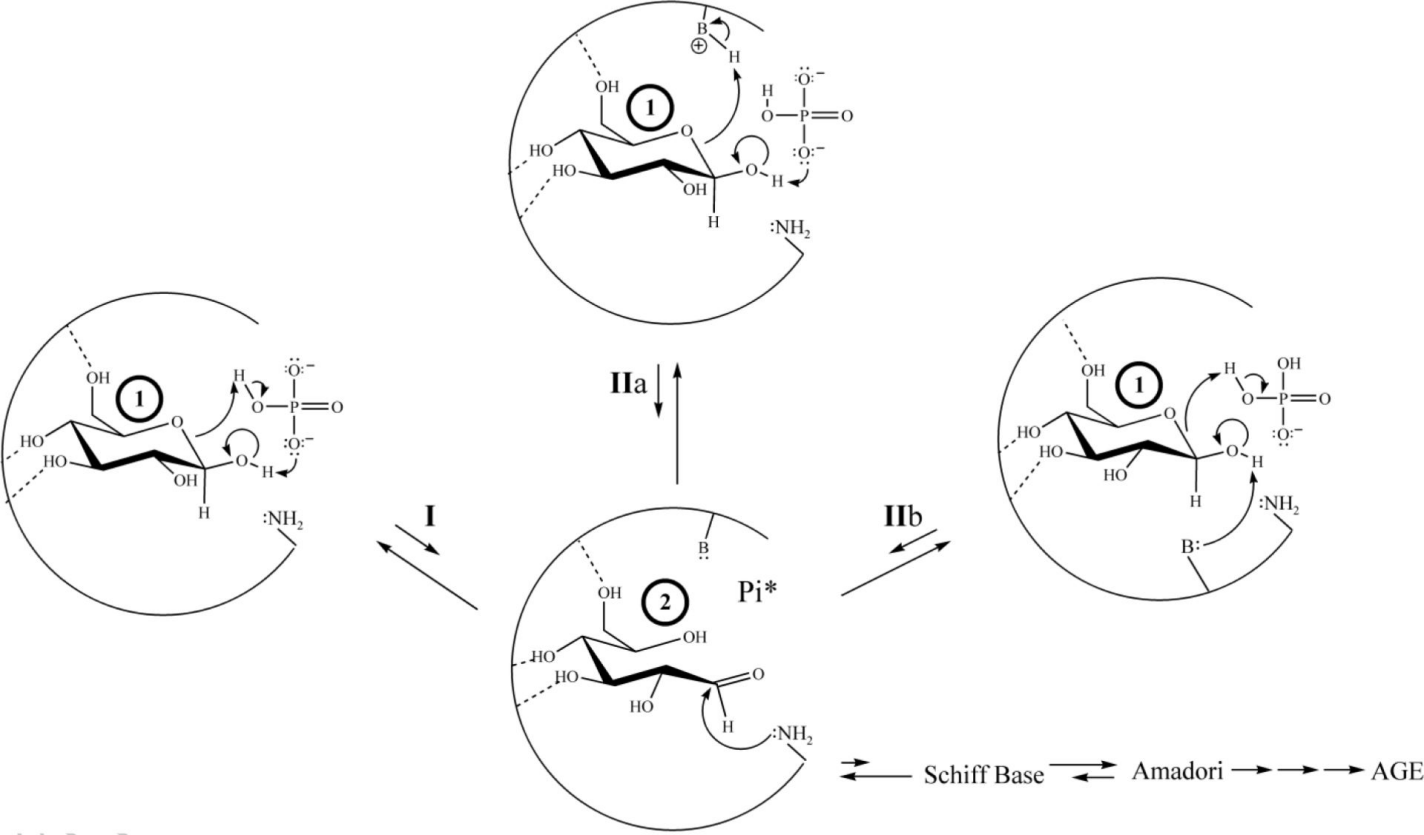
A 2-dimensional rendering of mechanisms for the concomitant binding of Pi (mono- or dianionic) and glucopyranose in HbA as determined by MOE computations. </P/>The partial circle represents the primary structure of the protein pocket; a basic amino acid residue (such as a neutral histidine, a carboxlyate, an amine R-NH_2_, etc) is rendered as -B:, an acidic amino acid residue (such as a histidine-H^+^, an R-NH_3_^+^of a lysine, etc.) is rendered as –B-H^+^; and the –NH_2_ represents the R-NH_2_ amine form of the N-terminal Val1, here acting as a nucleophile. Arrows within the partial circle (the protein pocket) represent bond making and bond breaking events.</P/>Two mechanistic classes involving concomitantly bound Pi and 1 that pass through or result in the formation of 2 are shown. The first mechanistic class (Mechanistic class I) is represented by transformation I. Two specific mechanisms within the second mechanistic class (Mechanistic class II) are represented by transformations IIa and IIb. For simplicity, only those amino acid residues and reagents that are directly involved in reaction are shown. The relative length of equilibrium arrows generally reflect the reversibility. Formation of a Schiff base is highly reversible, the Amadori intermediate is modestly reversible and AGE are irreversible. All variations of these mechanisms can occur with either α- or β-glucopyranose. </P/>Note: Pi* refers to the fact that Pi may either dissociate from, or remain within, the pocket as progrees in NEG proceeds.

**Figure 7.**
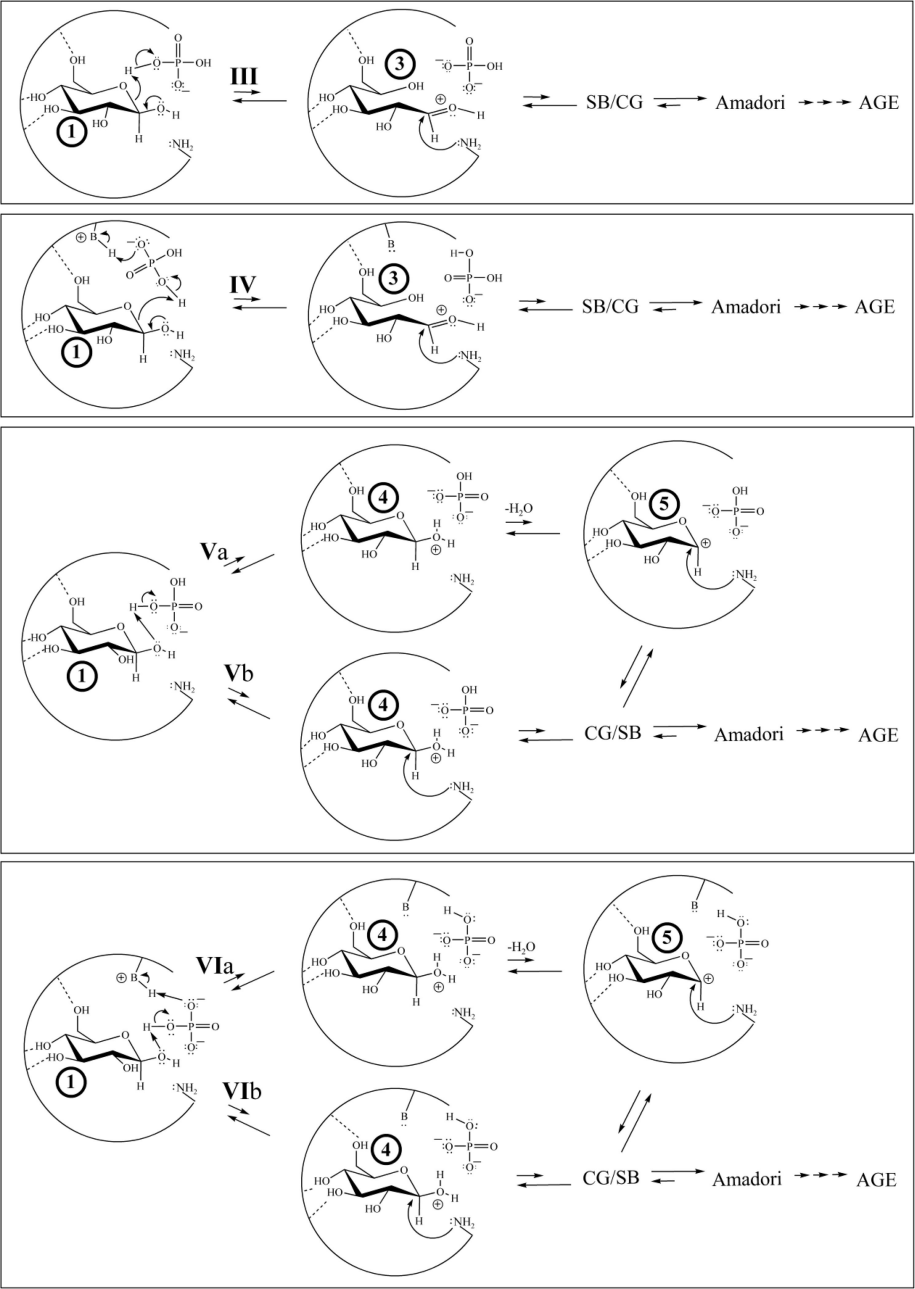
A 2-dimensional rendering of the four mechanistic classes for the concomitant binding of Pi and glucopyranose in HbA as determined by MOE computation that go through or generate transient electrophile 3 or 4 (4 may then generate 5). </P/>These mechanisms are possible for both α- and β-glucopyranose. Mechanistic classes IV, VIa and VIb are possible for either mono- or dianionic Pi, while mechanistic classes III, Va and Vb are for monoanionic Pi only. Mechanistic classes III and IV pass through transient electrophile 3. Mechanistic classes V and VI both generate 4. Intermediate 4, irrespective of how it is formed, can theoretically lose water to produce 5 (Vb and VIb). Classes III and IV initially form a protonated Schiff base (SB) and classes V and VI initially generate a cyclic glycosylamine (CG). The SB/CG designations (and the CG/SB designations) reflect that the Schiff base and the cyclicglycosylamine are in equlibrium with one another (Figure 2). The species listed first is the species initially formed. For all mechanisms, the only amino acids shown are those directly involved in the mechanism (either acid/base chemistry or to act as a nucleophile). The relative length of equilibrium arrows generally reflect the extent of reversibility.The formation of the protonated Schiff base is highly reversible while formation of the Amadori intermediate is modestly reversible. Progress through multiple steps to form various advanced glycation endproducts (AGE) is irreverisible. AGE are associated with diabetic complications.

Among dozens of energetic minima, six geometries representing six mechanistic classes with the potential to generate one (or more) of 2–5 were found (rendered in a classic arrow-pushing organic formalism for the sake of clarity in Figures 5, 6 and 7).

A mechanistic class is defined here as a general reaction process in which the identity of the reagents may vary, but the inherent bond-making/bond-breaking steps are the same.

### Computational assessment of geometrically feasible mechanisms for the water-assisted formation of 2–5 from a glucopyranose in HbA.

Similar to Pi, a single water molecule can concomitantly bind with either α- or β-glucopyranose in the HbA β-Val1 pocket and exists as an energetic minimum (Supplementary Figure 1). The generation of 2–5 via four mechanistic classes is predicted (Supplementary Figure 2). In addition, concomitant binding of water with each of the glucopyranoses takes place in the other three HbA pockets. In the Lys95 pocket, concomitant binding occurs with suitable geometry for reaction involving Lys95. Concomitant binding of water with each of the glucopyranoses occurs as local minima with suitable geometry to react with Lys61 within the Lys59/61/65/66 pocket. In the Lys17/120 pocket, concomitant binding occurs, but no suitable geometry for reaction is predicted.

### Computational assessment of geometrically feasible mechanisms of unassisted formation of 2–5 from a glucopyranose within the β-Val1 pocket of HbA.

If a glucopyranose binds alone, the only available acids and bases to generate transient electrophiles are nearby protein amino acid residues. Geometric computations show that a Lys R-NH_3_^+^ can act as an acid, while, in a concerted fashion, a neutral histidine can act as the base to generate transient electrophile 2. Once 2 is formed, a nucleophilic amino acid residue (e.g. β-Val1 or Lys if it is an R-NH_2_ amine) can then attack in a concerted process (Supplementary Figures 3 and 4).

## Discussion

Thirty years ago, investigators attributed the Pi-enhanced glycation of HbA (the Pi effect) to the covalent stages of the NEG process (Watkins et al., 1987; Kunika et al., 1989). This is not contested here. On the basis of the current work, additional Pi effects, specifically within the noncovalent stages of NEG (Figure 2), are posited. Glucose isomers must undergo modification while bound to a protein to generate the bound electrophiles necessary for efficient progress in NEG (Clark et al., 2013; Wang et al., 2013; Rodnick et al., 2017). A bound glucopyranose (Figure 3, Structure 1) can theoretically undergo modification to at least four transient electrophilic species. These include the ring-opened neutral glucose isomer (Structure 2), which is formed by protonation of the hemiacetal oxygen with concerted deprotonation of the anomeric OH. Structure 3, a protonated, ring-opened glucose, is formed by protonation of the hemiacetal oxygen by an acid. Structure 4, a protonated, ring-closed glucose, is generated by the protonation of the hydroxyl oxygen on the anomeric carbon. Structure 4 could react further by losing water to produce an oxocarbenium ion (Structure 5). Unlike 2 and 3, transient structures 4 and 5 remain ring-closed species. Each of these transient species (2–5) is more electrophilic than 1, and is likely to be much more reactive in NEG.

Once formed, 2–5 have three potential outlets. First, nucleophilic attack by an R-NH_2_ amino acid residue (such as β-Val1 or an internal Lys of HbA) can occur, which constitutes progress from the noncovalent to the covalent stages of NEG (Figure 2). Nucleophilic attack on either 2 or 3 would directly form a Schiff base, while nucleophilic attack on 4 or 5 would initially generate a cyclic glycosylamine. Note that the Schiff base and the cyclic glycosylamine are in equilibrium. As a second fate, 2–5 may exit the protein pocket without reacting with the nucleophile. As a third option, the modified species may revert back to bound 1 via rapid intramolecular processes and, once more, not proceed in NEG.

### Can Pi facilitate the formation of 2–5 from a glucopyranose?

The first experimental observation consistent with Pi-facilitated formation of any (or all) of 2–5 from a glucopyranose in aqueous solution (Table 1) is drawn from α-to-β glucopyranose inflection times. Both mono- and dianionic Pi facilitate the generation of electrophilic modified species from a glucopyranose (dianionic being the greater facilitator). This observation is consistent with previous studies of glucose mutarotation by Bailey, Fishman & Pentchev (1970). Second, α- to β-G6P inflection times indicate that modification of the original structure to an electrophilic species takes place more rapidly than does Pi facilitated α-glucopyranose to β-glucopyranose conversion. Interestingly, G6P is known to glycate β-Val1 of HbA much more efficiently than does glucose (Haney & Bunn, 1976). Third, G1P decreased α- to βglucopyranose inflection times by a factor of 10, likely because the phosphate on G1P is available for bimolecular reaction, playing a role similar to that of Pi.

In an effort to attain further mechanistic insight regarding Pi-facilitated α- to- β-glucopyranose interconversion, aqueous solutions at or near neutral pH with glucose:Pi molar ratios of 5:1 and 20:1, reflecting the relative intracellular concentrations of glucose in human erythrocytes (3–4 mmol/L, Santin et al., 2013) and Pi (0.5–0.8 mmol/L, Bevington et al., 1986) were prepared and inflection time experiments were performed. The inflection times for α- to- β-glucopyranose interconversion were much shorter than the benchmark (270 min), even when the HPO_4_^−2^ anion concentration is 1/20^th^ that of the sugar. That is, a rapid, reversible glucopyranose/Pi interaction occurred whereby interconversion is facilitated within the lifetime of a transient interaction, and this was true even at physiologically-relative concentrations.

### Validation that Pi-facilitated formation of reactive electrophiles is the result of Pi structural effect and not a pH effect.

Multiple reagents were evaluated for mechanistic purposes to discern whether inflection times are governed by effector reagent structure or whether there is a significant pH effect. From these studies, it is clearly a structural effect that is primarily manifest. First, large variation in inflection times were observed with different effector reagents at the same pH. Further, no trend exists in inflection time with pH (Table 1, Experiments 5–10). Second, at the common pH of 7.0, Bell et al. (1997) demonstrated significant reaction between glucose and amino acids in the presence of phosphate buffer but not in the presence of citrate buffer. Thus, *at identical pH, different buffers promote the NEG process to different degrees --- a structural effect rather than a pH effect.* Third, the ^31^P chemical shift data is consistent with a reversible adduct between dibasic Pi and glucopyranose. Fourth, as computational validation (ab initio HF631G**) for the formation of the bridging adduct is 30 kcal/mol exothermic relative to isolated starting materials. Together, these data suggest that the primary structural effect of Pi is to bridge with a glucopyranose, enabling the generation of 2.

While this data supports the contention that Pi does facilitate the formation of modified forms of glucopyranose, they do not directly enable the assignment of 2–5. Further, these results are relevant only if analogous reactions can take place between a Pi and a glucopyranose while concomitantly bound in a protein pocket.

### Can Pi concomitantly bind with glucopyranose isomers in HbA?

Computational modeling (Table 2) predicts that exothermic, reversible binding of mono- and dianionic Pi with 1 occurs in known HbA glycation pockets in competition with reversible single-molecule binding of Pi and glucopyranose in those same pockets. X-ray crystallographic validation of concomitant binding of glucopyranose and Pi has been documented for human serum albumin (Wang et al., 2013).

### Are there suitable geometries for the Pi-facilitated formation of 2–5 from a glucopyranose while concomitantly bound in the β-Val1 pocket of HbA?

*If so, what mechanistic possibilities exist?* Computations predict an array of geometries of noncovalently bound Pi, glucopyranose, and proximate amino acid residues suitable for the formation of 2–5. Six mechanistic classes of reactions are geometrically predicted (Figures 6 and 7).

In mechanistic class I, Pi (mono- or dianionic) is geometrically able to bridge the bound glucopyranose (in which the Pi has two points of molecular interaction with the glucopyranose) by protonating the hemiacetal oxygen while deprotonating the anomeric OH in a concerted process, passing through 2 (Figure 6). Support for Pi participation in a concerted acid/base reaction via a Pibridging interaction comes from HF-631G^**^ *ab initio* computations (gas-phase). Each of the glucopyranose isomers (1) are predicted to bridge with either mono- or dianionic Pi. Moreover, this mechanism is analogous to that proposed for the mutarotation of glucose in aqueous solution at physiological pH in which water is the bridging agent (Silva, E. da Silva & C. da Silva, 2006).

In mechanistic class II, Pi can act as either acid or base in conjunction with a proximate amino acid residue (Figure 6), which also reacts with the glucopyranose (1) and again passes through 2. The species acting as the base can be a Lys R-NH_2_, dianionic Pi, or neutral His. The species serving as the acid can be a Lys R-NH_3_^+^, monoanionic Pi, or His-H^+^. Regardless of the specific acid or base, this mechanism passes through transient electrophile 2. Each variation in this mechanistic class is reasonable because analogous mechanisms (both stepwise, involving discrete intermediates and concerted, not involving discrete intermediates) are observed for the enzyme galactose mutarotase (Thoden et al., 2003). Irrespective of how 2 is generated or whether nucleophilic attack takes place as 2 is formed (concerted) or after (stepwise), a Schiff base can be generated. The Schiff base can then revert back to 1 or it can progress through NEG.

In mechanistic class III, the hemiacetal oxygen of a bound glucopyranose (1) is protonated by monoanionic Pi, His-H^+^, or Lys R-NH_3_^+^ (Figure 7). This process passes through transient electrophile 3, consistent with related chemistry proposed by Wang et al. involving HSA (Wang et al., 2013). In mechanistic class IV, Pi is protonated by a Lys R-NH_3_^+^ or His-H^+^, which then protonates the hemiacetal oxygen of 1, again passing through 3 in a concerted fashion (Figure 7). Johansen, Kiemer & Brunak (2006) have shown that a carboxylate is capable of abstracting the proton of an R-NH_3_^+^ amino acid residue in a concerted process in conjunction with nucleophilic attack, making it likely that Pi (mono- or dianionic) is also a sufficient base for this process.

In mechanistic class V, the geometries of various local minima enable the anomeric OH of a bound 1 to be protonated by monoanionic Pi, such that progression through transient electrophile 4 is possible (Figure 7). Once more, either concerted (Numao et al., 2002) or stepwise processes (Qian, 2012) are theoretically possible. Mechanistic class VI also involves the protonation of the anomeric OH of bound 1 by monoanionic Pi, but here Pi undergoes concerted protonation by a Lys R-NH_3_^+^ or His-H^+^ (Figure 7). This process also passes through 4 and retains the original charge state of the Pi. Whether formed by mechanism V or VI, 4 can be attacked by a nucleophile to generate the cyclic glycosylamine that can continue through NEG. Another potential fate is for water to detach from 4 as a leaving group prior to nucleophilic attack in a stepwise process, passing through 5 (Figure 7) and once more proceeding to a cyclic glycosylamine.

### Can water facilitate the formation of bound 2–5 from a bound glucopyranose?

A single water molecule can concomitantly bind in the β-Val1 pocket with either α- or β-glucopyranose and facilitate the formation of 2–5 via four mechanistic classes of reaction (fully described in Supplementary Figure 2) such that HbA1c can be generated. In each of these mechanisms, the β-Val1 R-NH_2_ serves a single role as the nucleophile. Concomitant binding of water and glucopyranose in either the Lys95 pocket or the Lys59/61/65/66 pocket possess geometries suitable for reaction involving Lys95 and Lys61, respectively. However, the amino acid residue to be glycated (Lys95 or Lys61) exists initially as an R-NH_3_^+^ and thus must first act as an acid to protonate water, which must then protonate and ring open 1. The resulting R-NH_2_ amino acid can then theoretically react as a nucleophile. Such mechanisms, where a single amino acid residue plays two roles, are referred to here as dual-role mechanisms. Lys95 is not an experimentally observed glycation site (Delpierre, Vertommen, Communi, Rider & Schaftingen, 2004), indicating that dual-role mechanisms are less viable than mechanisms that do not require a dual-role. As such, these types of mechanisms are predicted to be minor processes.

### Can 2–5 be generated in the HbA β-Val1 pocket without the concomitant binding of an effector reagent?

An effector reagent (ER) is any molecule that may facilitate or inhibit one or more mechanistic steps in NEG. Computations predict that a Lys R-NH_3_^+^ can act as an acid, while, in a concerted fashion, a neutral histidine acts as a base to generate 2 (Supplementary Figure 3a and 4) which is attacked by the β-Val1 R-NH_2_ (in a concerted or stepwise fashion) as the first covalent step towards the ultimate generation of HbA1c. That histidine can abstract a hydroxyl proton in a concerted charge relay system is reasonable because far less basic species (e.g. carboxylate anions) are capable of this reaction in related systems (Zhang et al., 2001; Thoden et al., 2003; Wang et al., 2013; Stergiou, Foukis, Gkini, Bieth & Papamichael, 2016; Vladimirova et al., 2016). Further, examples of His-H^+^ and β-Val1 or Lys R-NH_3_^+^ residues acting as acids in concerted mechanisms have been reported (Zhang et al., 2001; Wang et al., 2013).

Glycation in the absence of Pi (or water, or any other ER) is much less likely than glycation in the presence of Pi (or other ER). First, less than 10% of the computationally determined local minima meet the geometric requirement for this reaction. Therefore, the likelihood of HbA1c formation from 1 bound alone in the absence of an effector reagent is much lower than in the presence of an effector reagent (where 27% of minima can lead to glycation at Val1). This is significant in potentially explaining a pre-Amadori rate-determining Pi effect. Only one mechanism is predicted when glucopyranose binds alone in the β-Val1 pocket, whereas in the same pocket a glucopyranose in the presence of Pi enables six mechanistic categories comprising ca. 50 mechanisms. The likelihood of arriving at the geometry for the single mechanism for 1 alone is much lower than the probability of attaining suitable geometry for any of the ER-assisted mechanisms. Finally, the single mechanistic possibility can proceed only if the histidine is neutral (which is not always the case).

### *What is the impact of mechanistic diversity on site selectivity in the early, noncovalent stages of HbA glycation?* The data addressing this question are summarized in Table 3.

Based upon the computational assessment of mechanistic possibilities in the β-Val1 pocket, we predict that Val1 will experience a high degree of glycation. This is in part due to its inherently high percentage as the R-NH_2_ form under physiological conditions (Matthew, Hanania & Gurd, 1979), making it a readily-available nucleophile. Further, Val1 is predicted to be in proximity to a relatively high concentration of bound 2–5 due to effective concomitant binding of both glucopyranose isomers with each species of Pi (especially dianionic Pi). In fact, Pi effects in the Val1 pocket are more pronounced than in any of the other HbA β-chain pockets. All of these factors contribute to the ability of β-Val1 to progress in NEG. While the glucopyranoses bind near Lys82, Lys82 is predominately in its R-NH_3_^+^ form (Matthew et al., 1979) and hence, is non-nucleophilic and not able to react. That said, concomitant binding events of 1 with either species of Pi occur, and thus Pi can facilitate deprotonation to the nucleophilic amine. However, the Lys82 R-NH_2_ must then react as a nucleophile in a dual-role mechanism before 2–5 either revert back to 1 or dissociate from the pocket. This temporal demand may explain why little glycation at Lys82 is observed. No geometries predict a Lys8 glycation event. Lys132 and 144 [both predominately RNH_3_^+^ ammonium ions (Matthew et al., 1979)] are also not predicted to undergo significant glycation, because neither has the proper geometry to react during the binding of 1 and neither species of Pi binds to these residues in a manner that could lead to deprotonation and the generation of a nucleophile.

In the Lys59/61/65/66 pocket, glucopyranose alone binds near each lysine residue, although Lys59 and Lys66 are more accessible. However, like Lys82, these amino acid residues are predominately R-NH_3_^+^ ammonium ions (Matthew et al., 1979) and are unlikely to proceed in NEG. Glucopyranose concomitant binding with Pi occurs near Lys59, Lys61 and Lys65 but not within reacting distance to Lys66. Overall, little glycation within this pocket is predicted.

Lys17 in the Lys17/120 pocket binds only α-glucopyranose with dianionic Pi. Lys120 binds neither glucopyranose isomer nor either Pi species independently, and no concomitant binding with dianionic Pi occurs with suitable geometry to react. Thus, little glycation of Lys120 or Lys17 is predicted.

In the Lys95 pocket, no glycation is computationally predicted because neither glucopyranose isomer nor either Pi species independently binds near Lys95 and no concomitant binding with dianionic Pi occurs with suitable geometry to react at this residue. The water-assisted Lys95 reaction is unfavorable as well, owing to it being a dual-role mechanism.

There is experimental support for each of our computational predictions regarding site selectivity for HbA glycation on the β-chain (Shapiro, McManus, Garrick, McDonald & Bunn, 1979; Zhang et al., 2001; Delpierre et al., 2004; Wang et al., 2013). Overall, β-Val1 is the most glycated residue of HbA (reviewed in Delpierre, 2004), consistent with our computations. Lys82 (also in the β-Val1 pocket) however, is predicted to undergo limited glycation which is consistent with *in vivo* glycation for HbA (Zhang et al., 2001; Delpierre et al., 2004). This is especially interesting because when glyceraldehyde is used as a glycation surrogate, Lys82 glycates readily (Nacharaju & Acharya, 1992; Ito et al., 2011). Glyceraldehyde does not undergo an NEG process, but instead follows a nonenzymatic covalent protein modification (NECPM) mechanism and cannot be compared directly to NEG (Rodnick et al., 2017). Zhang et al. reports that Lys8 combined with Lys17 is ca. 1% of total glycation (Zhang et al., 2001). Our predictions are in agreement. Delpierre et al. (2004) report Lys132 and 144 as the two lowest glycating residues, while Zhang reports as much as 6% glycation of Lys132. Zhang et al. (2001) reports the combined glycation of Lys61/65/66 as 9% of all glycation events. Lys66 is reported for modest glycation by Shapiro and Delpierre, neither of whom indicates glycation for Lys65 (Shapiro et al., 1979; Delpierre et al., 2004). Our binding and geometric assessments predict that these residues would be glycated to a low degree. Lys95 is experimentally a non-glycating residue (Shapiro et al., 1979; Zhang et al., 2001; Delpierre et al., 2004), precisely what we predict. The consistency between computationally-predicted site selectivity as determined by chemistry in the non-covalent stages and experimental observations suggests that diverse Pi effects in the non-covalent stages may impact the site selectivity of NEG for HbA (Table 3).

Which mechanism prevails in a given HbA pocket is based on multiple factors. One factor is the identity of the species bound in a given pocket (α- or β-glucopyranose, mono- or dianionic Pi, and/or water; concomitant or single species binding). Another is the specific geometries of the bound species relative to one another and to amino acid residues. The relative extent of mono- versus dianionic Pi is likely to impact the nature and extent of NEG because dianionic Pi facilitates many more mechanisms, while monoanionic Pi undergoes more exothermic concomitant binding with 1 and binds to more sites (Table 2). Further, the identity and number of potentially acidic, basic, and/or nucleophilic amino acid residues are factors. Specifically, the presence of proximate N-terminal valine residues and lysine R-NH_2_ versus R-NH_3_^+^ affect mechanistic possibilities. The number, geometric availability, charge state, and resulting pKa of nearby histidines and carboxylates are also factors in determining which specific mechanisms are manifest. For a more complete assessment of these mechanisms, future studies should consider the persistence of reagents in various protein pockets, a concept referred to as residence time (Tummino & Copeland, 2008).

Site selectivity for NEG will likely be governed by the same variables that govern mechanisms. We propose the β-Val1 pocket of HbA is a clinically-relevant “hot spot” because it can support multiple effector reagents and facilitates many more mechanistic possibilities than any other site. Further, β-Val1 is in an R-NH_2_ nucleophilic form within these variable mechanisms with a much higher probability than the competing lysine residues, which are most often non-nucleophilic R-NH_3_^+^ ions (Matthew et al., 1979). Within the β-Val1 pocket, glycation of Val1 leading to HbA1c prevails over glycation of Lys82 and other nearby lysine residues in the same pocket based upon these same mechanistic arguments. The scope of this paper does not include the impact of the organic phosphate 2,3-bisphosphoglycerate (2,3BPG), which is well known to bind to the same pocket (Arnone & Perutz, 1974). The effect of 2,3-BPG on the degree of mechanistic diversity, and its impact on site selectivity in the glycation of HbA is a focal point of our follow-up work.

Finally, we posit that HbA1c formation (and protein glycation in general) is slow because the predominant species bound (a glucopyranose, 1) binds in an unreactive form that must undergo reversible modification while bound. Further, the likelihood of the proper geometry for 1 to proceed in NEG in the absence of an ER is low. Facilitation by an ER enhances the overall rate, yet is a low probability event. First, concomitant binding events of a glucopyranose with an ER (i.e. Pi or water) are low probability events. Second, a suitable geometry to react is required for ER participation and these events are predicted to be rare (many more geometries are computationally observed that do not possess the proper geometry to react relative to those that do). Third, the resulting bound modified glucose electrophile must react with a nucleophilic amino acid residue before either reversion to 1 or dissociation from the pocket occurs. Finally, a suitable nucleophile must be available with proper geometry within the lifetime of the electrophile. The requirement of meeting all four criteria simultaneously may contribute to why the glycation of HbA to HbA1c is a slow process.

## Conclusions

In addition to Pi effects in the covalent stages of the NEG process, Pi effects are manifest in the early, noncovalent stages of NEG. Pi acts as an effector reagent by concomitantly binding with 1 and is predicted to react, while bound to HbA, to generate an array of transient electrophiles via multiple mechanisms. Six mechanistic classes and ca. 50 potential mechanisms, many of which may be concerted or stepwise, are predicted. Which mechanism prevails in a given HbA pocket is based 1) the identity of the species bound in a given pocket (α- or β-glucopyranose, mono- or dianionic Pi, and/or water; concomitant or single species binding); 2) the specific geometries of the bound species relative to one another and to amino acid residues; 3) the relative extent of mono- versus dianionic Pi; and 4) the identity and number of potentially acidic, basic, and/or nucleophilic amino acid residues. The new Pi effects reported upon here in the non-covalent stages of the NEG process can help explain both HbA glycation site selectivity and the relatively slow rate of NEG.

## Experimental Procedures

### NMR Experiments:

All ^1^HNMR experiments were performed on a JEOL ECX-300 spectrometer. Reagents were all purchased commercially and used without further purification. Inflection time measurements were made by preparing a 1:1 molar equivalent mixture of the α-glucopyranose to effector reagent at a composite concentration of 140 mmol/L in D_2_O. We also measured the time for αG6P inflection to β-G6P in absence of any effector reagent except D_2_O. Reactions were followed as a function of time (ca. 5 min being the earliest time point based upon getting the NMR tube loaded, locked, and shimmed). From there, data was collected every 2–15 min, depending on the reaction progression. The doublet at 5.06 ppm for the proton on the anomeric carbon of the α-glucopyranose isomer diminishes with time as the doublet at 4.47 ppm for the proton on the anomeric carbon of the β-glucopyranose isomer correspondingly increases with time. The time that is required for the integration of the measured signals for the protons for the α-and β-glucopyranose isomer (at 5.06 and 4.47 ppm) to become equal is defined as the inflection time. As inflection times decrease, the rate of passage through the necessary transient electrophile (ring-opened isomer, Figure 1) increases. An identical process was utilized to determine the inflection for α-G6P to β-G6P (following the doublet for the proton on the anomeric carbon of each species). These model reactions were used to assess the impact of an effector reagent to facilitate transient electrophile formation from a glucopyranose. This comparison between effector reagents was designed to give a qualitative understanding of relativistic effector reagent impact. The solution pH of each of the samples subjected to NMR analysis was determined with a pH meter. ^31^PNMR experiments were designed to follow chemical shift differences in mono- and/or dianionic Pi, as well as G6P, in their interactions with glucopyranoses. Reaction conditions and relative concentrations were identical to those used in our ^1^HNMR experiments. All ^31^PNMR were conducted at 20°C with chemical shifts measured against external standard phosphoric acid.

### Computational substrates.

Protein/substrate computations utilized Molecular Operating Environment (MOE, ver. 2014.9, Chemical Computing Group Inc., Montreal). The structures utilized in the computations for protein-substrate interaction were as follows: a) the fully oxygenated HbA crystal tetramer 1GZX was obtained from the RCSB PDB (www.pdb.org/pdb/home/home.do), b) the α- and β-glucopyranose isomers were obtained from Heterocompound Information Centre, Uppsala, (HICUP, http://xray.bmc.uu.se/hicup/) and c) mono- and dianionic Pi and water were built with The PyMOL Molecular Graphics System (ver. 1.5.0.4 Schrödinger, LLC, www.pymol.org/) or made within MOE. Ab initio computations of substrate Pi adduct formation were performed in Spartan, version 1.0.3 (Wavefunction Inc. Irvine, CA USA).

#### Computational modeling to assess the binding of substrates to HbA.

Within MOE, the HbA β-chain utilized was modified such that the Val1 was an R-NH_2_ amine. All lysines were R-NH_3_^+^ ammonium ions. Single substrate experiments utilized the complete HbA protein. Structures that were exothermic beyond the limit of −2.5 kcal/mol (the binding exothermicity of water) were analyzed, with a cutoff distance of 5Å established for effective reaction within protein pocket(s) (Bobadilla, Nino & Narasimhan, 2004; Ito et al., 2011). For concomitant binding studies, a glucopyranose was tether-docked in the pocket of interest and then a second substrate (i.e. Pi or water) was then inserted and energy minimized.

## Supplementary Material

1

## Figures and Tables

**Table 1.** Time-dependent ^1^HNMR data for α- to β-glucopyranose inflection through the ring-opened glucose isomer 2 in D_2_O at ~20°C in the absence or presence of various physiological anions (serving as effector reagents, ER).

Experiment	Sugar	Sugar Equivalency	Effector Reagent(s)	Effector Reagent Equivalency	pH	Inflection Time(min)
1	α-Glucose	1	D_2_O	-	5.5	270
2	α-Glucose	1	HPO42-	1	9.1	8
3	α-Glucose	1	H2PO4-	1	4.5	215
4	α-G6P	1	-	-	4.2	<5*
5	α-Glucose	1	Histidine^#^	1	4.2	280
6	α-Glucose	1	Taurine^#^	1	5.1	150
7	α-Glucose	1	Metformin^#^	1	6.4	230
8	α-Glucose	1	Lactate^#^	1	7.1	190
9	α-Glucose	1	2,3-BPG^#^	1	7.2	46
10	α-Glucose	1	HCO3-	1	8.4	28
11	α-Glucose	1	HPO42- H2PO4-	Composite = 1	6.4	87
12	α-Glucose	1	HPO42- H2PO4-	Composite = 1	6.8	70
13	α-Glucose	1	HPO42- H2PO4-	Composite = 1	7.0	30
14	α-Glucose	1	HPO42- H2PO4-	Composite = 1	7.4	12

* Inflection of α-glucose-6-phosphate (α-G6P) occurs faster than can be measured.

# These effector reagents have variable charge states based upon pH.

**Table 2.** Computational assessment of α- and β-glucopyranose concomitant binding with Pi to the fully-protonated β-chain of oxygenated HbA organized by potential glycation sites, binding exothermicities, and proximate amino acid (AA) residues.

Glucopyranose	Phosphate	Binding Energy (ΔH, kcal/mol)	Proximate Amino Acids
Α	Pi-2	−3.6 to −3.2	Val1, Lys8, Lys17, Lys 61, Lys 82
Β	Pi-2	−3.8 to −3.2	Val1, Lys8, Lys82, Lys144
Α	Pi^-^	−6.4 to – 5.0	Val1, Lys17, Lys59, Lys61, Lys82, Lys95, Lys120, Lys144
Β	Pi^−^	−6.6 to −5.1	Val1, Lys8, Lys17, Lys59, Lys61, Lys82, Lys95, Lys120, Lys132, Lys144

This data was generated through the assessment of distances between the electrophillic site on the bound glucopyranose and nearby nucleophillic sites on the amino acid residues as measured in MOE (Molecular Operating Environment). A distance deemed proximate is a distance, given protein flexibility, where by reaction can theoretically occur. Amino acid residues within 5Å were considered proximate. These measurements were made with the assumption that a 3–5Å variation within the binding environment is possible (Bobadilla et al., 2004; Ito et al., 2011). Note: Pi^−^ is monoanionic Pi and Pi^−2^ is dianionic Pi.

**Table 3.** Summary comparison of computational predictions for HbA β-chain glycation site selectivity versus experimentally-observed HbA site selectivity both *in vivo* and *in vitro*. The current computations (column 1) reflect only those interactions within the non-covalent stages of the NEG process with the intent to discern whether noncovalent interactions between a glucopyranose and amino acid residues in an HbA pocket and/or inorganic phosphate (mono- or dianionic) can explain experimentally observed NEG site selectivity (as measured by HbA Amadori products, columns 2–4).

Computational Prediction of Noncovalent Interactions and Likelihood for Progress in NEG	Experimental Observation of Amadori Formation		
	*In vivo* Glycation Shapiro et al. (1979)	*In vivo* Glycation Zhang et al. (2001)	*In vitro* Glycation Delpierre et al. (2004)
Val1 = high Lys 8^#^ = none Lys82* = low/none Lys132^#^ = low/none Lys144^#^ = low/none	Val1 = high Lys 8 = none Lys82 = none Lys132 = none Lys144 = none	Val1= high Lys 8 = low/none Lys82 = none Lys132 = low/none Lys144 = low/none	Val1= detected Lys 8 = not detected Lys82 = not detected Lys132 = detected Lys144 = detected
Lys17^^^ = low/none Lys120^^^ = low/none	Lys17 = low Lys120 = none	Lys17 = low/none Lys120 = none	Lys17 = detected Lys120 = not detected
Lys59* = low/none Lys61^^^ = low/none Lys65^^^ = low/none Lys66^^^ = low/none	Lys59 = none Lys61 = none Lys65 = none Lys66 = low	Lys59 = none Lys61 = low/none Lys65 = low/none Lys66 = low/none	Lys59 = detected Lys61 = not detected Lys65 = not detected Lys66 = detected
Lys95^#^*^ = none	Lys95 = none	Lys95 = none	Lys95 = not detected

To proceed from the non-covalent to the covalent stages of NEG, multiple criteria must be met. A glucopyranose, 1, must bind exothermically in a HbA pocket and then be converted to any of the modified, electrophilic glucose species 2–5 through acid/base chemistry with amino acid residues and/or concomitantly bound Pi. The bound electrophile (any of 2–5) must be within reacting distance (5Å) of a suitable nucleophile, which must then attack before the modified glucose electrophile reverts back to 1 or dissociates from the pocket. Within column 1, the categories of ‘high,’ ‘low/none,’ and ‘none’ indicate the extent to which these simultaneous criteria are met. The superscript notations on amino acid residues indicate the specific criterion that was not met for that residue.

# A glucopyranose, 1, is not computationally predicted to bind within interacting distance to react with the designated residue;

* Requires a dual role mechanism whereby an R-NH_3_^+^ must firstly be deprotonated to and R-NH_2_ which must then react as a nucleophile; a low probability event;

^ Little or no concomitant binding of Pi/glucopyranose observed near residue or concomitant binding occurs but a suitable geometry is not observed.

## ABBREVIATIONS

2,3-BPG: 2,3-bisphosphoglycerate
AGE: advanced glycation endproducts
G1P: glucose-1-phosphate
G6P: glucose-6-phosphate
HbA: adult hemoglobin
HbA1c: adult hemoglobin A1c
HSA: human serum albumin
MOE: Molecular Operating Environment
NEG: nonenzymatic glycation
NMR: nuclear magnetic resonance
Pi: inorganic phosphate
