# Supplementary material for: Potential roles of inorganic phosphate on the progression of initially bound glucopyranose toward the nonenzymatic glycation of human hemoglobin: mechanistic diversity and impacts on site selectivity
Source: Cogent Biol. Author manuscript; Available in PMC 2019 Mar 25. (PMC6433390; doi:10.1080/23312025.2018.1425196)
Supplement: 1 [file NIHMS1505321-supplement-1.pdf]

Supplementary Figure 1. Representative energetic minima whereby water and a β-glucopyranose concomitantly bind in the β-Val1 pocket of HbA. For visual clarity, only those residues needed to illustrate the geometrically viable mechanisms are shown.

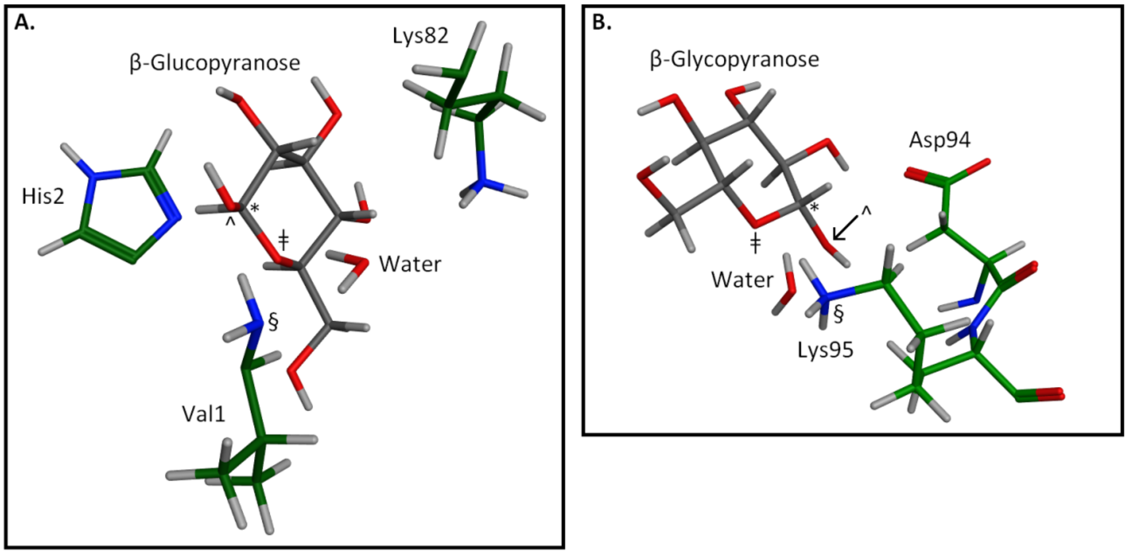

* anomeric carbon; ^ anomeric OH; ‡ hemiacetal oxygen; § nucleophile. Red is oxygen; blue is nitrogen; pink is phosphorous; gray are glucopyranose carbons; green are amino acid residue carbons; white are hydrogens.

From this geometry, four distinct mechanisms are possible. In one mechanism the water bridges the glucopyranose, and, in a concerted fashion, protonates the hemiacetal oxygen (dist. = 1.74Å) while deprotonating the anomeric OH (dist = 3.86Å) as Val1 attacks the incipient anomeric carbonyl (dist. = 3.89Å) on route to a protonated Schiff base (equating to Figure 5, mechanism I). In a second mechanism, the Lys82 protonates water (dist. = 3.96Å) as the water acts as an acid to protonate the hemiacetal oxygen (dist. = 1.74Å) and His2 deprotonates the anomeric OH (dist. = 4.74Å) as a nucleophilic Val1 attacks the resulting electrophilic anomeric carbon of the in a concerted fashion (dist. = 3.89Å) on route to a protonated Schiff base (equating to Figure 5, mechanism I). Alternatively, the water can act as a base to deprotonate the anomeric OH (dist = 3.86 Å) as His2 deprotonates water (dist. = 5.39Å) and Lys82 protonates the hemiacetal oxygen (dist = 4.55Å) as a nucleophilic Val1 attacks the resulting electrophilic anomeric carbon of the in a concerted fashion (dist. = 3.89Å) on route to a protonated Schiff base (equating to Figure 5, mechanism I). In a third mechanism, water protonates the anomeric OH (dist. = 3.06 Å) as Lys82 protonates water (dist. = 3.96 Å), making the anomeric carbon electrophilic and generating water as a leaving group as the Val 1 attacks as a nucleophile (dist. = 3.89Å) on route to a cyclic glycosylamine (equating to Figure 6, Mechanism VIa). In a fourth mechanism, the Lys82 protonates water (dist. = 3.96Å) as the water protonates the hemiacetal oxygen of the glucopyranose (dist. = 1.74Å), making the anomeric carbon electrophilic as Val1 attacks as a nucleophile in a concerted fashion (dist. = 3.89Å), generating 3 (equating to Figure 6, Mechanism IV) on route to a protonated Schiff base.

ACCEPTED MANUSCRIPT

From this geometry, three distinct mechanisms are possible. In the first mechanism, the water protonates the hemiacetal oxygen (dist. =1.73 Å) as Lys95 protonates water (dist=2.66 Å) and Asp94 deprotonates the anomeric OH (dist= 3.62Å) as the Lys95 attacks the incipient anomeric carbonyl in a concerted fashion (dist. = 5.08 Å) on route to a protonated Schiff base (equating to Figure 5, mechanism I). In a second mechanism, the water protonates the anomeric OH (dist. = 2.78 Å) as Lys95 protonates water (dist. = 2.66 Å), making the anomeric carbon electrophilic and generating water as a leaving group as the Lys95 attacks as a nucleophile (dist. = 5.08Å) on route to a cyclic glycosylamine (equating to Figure 6, Mechanism VIa). In a third mechanism, the Lys95 protonates water (dist. = 2.66Å) as the water protonates the hemiacetal oxygen of the glucopyranose (dist. =1.73Å), making the anomeric carbon electrophilic as the Lys95 attacks as a nucleophile in a concerted fashion (dist. = 5.08 Å), generating 3 (equating to Figure 6, Mechanism IV) on route to a protonated Schiff base. In all the aforementioned mechanisms, Lys95 must play a dual-role; It must first act as an acid and donate a proton then act as a nucleophile before either re-ring closure of the transient glucose electrophile or substrate disassociation occurs. Such mechanisms, while geometrically viable have temporal considerations making reaction less likely than mechanisms where a single species does not need to play a dual role.

ACCEPTED MANUSCRIPT

Supplementary Figure 2. A 2-dimensional rendering of the mechanistic classes for the concomitant binding of water and a glucopyranose 1 in HbA as determined by MOE computations.

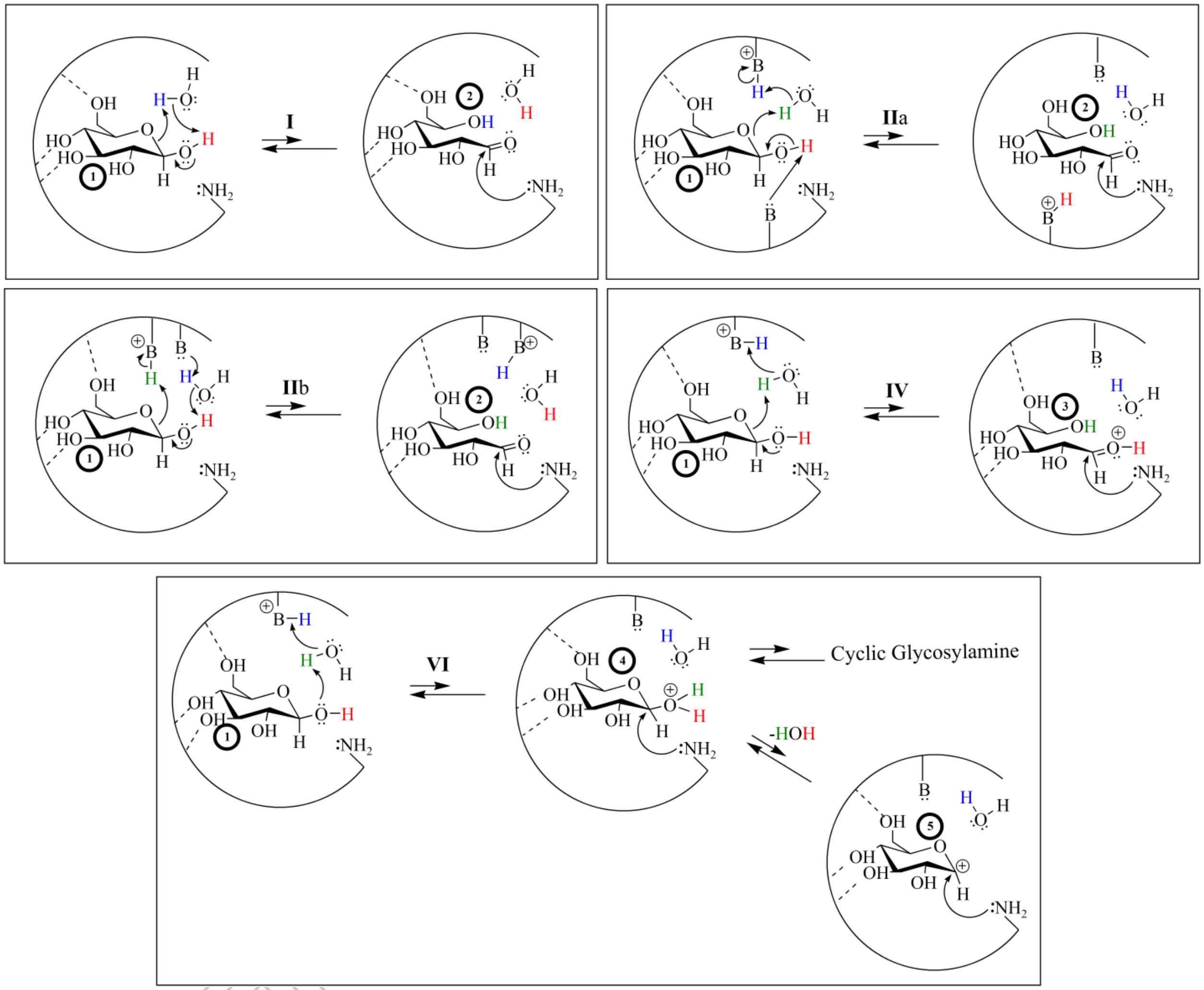

Transient electrophiles (2-5), once formed, can theoretically proceed to a protonated Schiff base or a cyclic glycosylamine (which is in equilibrium with the Schiff base). The protonated Schiff base can then proceed to an Amadori intermediate and, finally, to AGE. In mechanistic class I, a single water molecule acts as the bridging agent to deprotonate the anomeric OH and protonate the hemiacetal oxygen in a concerted process, generating 2; this reaction is comparable to mechanistic class I for glucopyranose and Pi interactions (Figure 5, Mechanism I). Mechanistic class IIa involves a basic amino acid residue (His or Lys R-NH_2) abstracting the anomeric OH of the bound 1, initiating ring-opening as the hemiacetal oxygen abstracts a proton from water while water abstracts a proton from a proximate acidic amino acid (His-H^+ or Lys R-NH_3^+). In mechanistic class IIb, an amino acid residue (His or Lys R-NH_2) abstracts a proton from water, as the water abstracts the anomeric OH, initiating the glucopyranose, 1, to ring-open. The hemiacetal oxygen then abstracts a proton from a nearby His-H^+ or Lys R-NH_3^+ in a concerted fashion. Both IIa and IIb generate transient electrophile 2 and these mechanisms are analogous to

mechanistic classes IIa and IIb for glucopyranose and Pi interactions (Figure 5, Mechanism IIa and IIb). In mechanistic class IV, a lone pair of electrons from the anomeric hydroxyl group begins to form a π–bond initiating ring-opening as the hemiacetal oxygen abstracts a proton from water, as water abstracts a proton from a nearby amino acid (His-H^+ or Lys R-NH_3^+), generating 3. This mechanistic class is similar to mechanistic class IV for glucopyranose and Pi (Figure 6, Mechanism IV). In mechanistic class VI, the oxygen of the anomeric hydroxyl begins to abstract a proton from water, as water abstracts a proton from a proximate His-H^+ or Lys R-NH_3^+ amino acid, generating 4. Intermediate 4 can either be attacked by a nucleophile and progress to the cyclic glycosylamine (which is in equilibrium with the Schiff base), or water can depart from 4 to generate the oxocarbenium ion, 5, which is then subject to nucleophilic attack. This class is akin to mechanistic class VI for glucopyranose and Pi reactions (Figure 6, Mechanism VI).

ACCEPTED MANUSCRIPT

Supplementary Figure 3. A representative energetic minimum whereby no effector reagent is involved. Here the acids/bases that facilitate transient electrophile formation are amino acid residues.

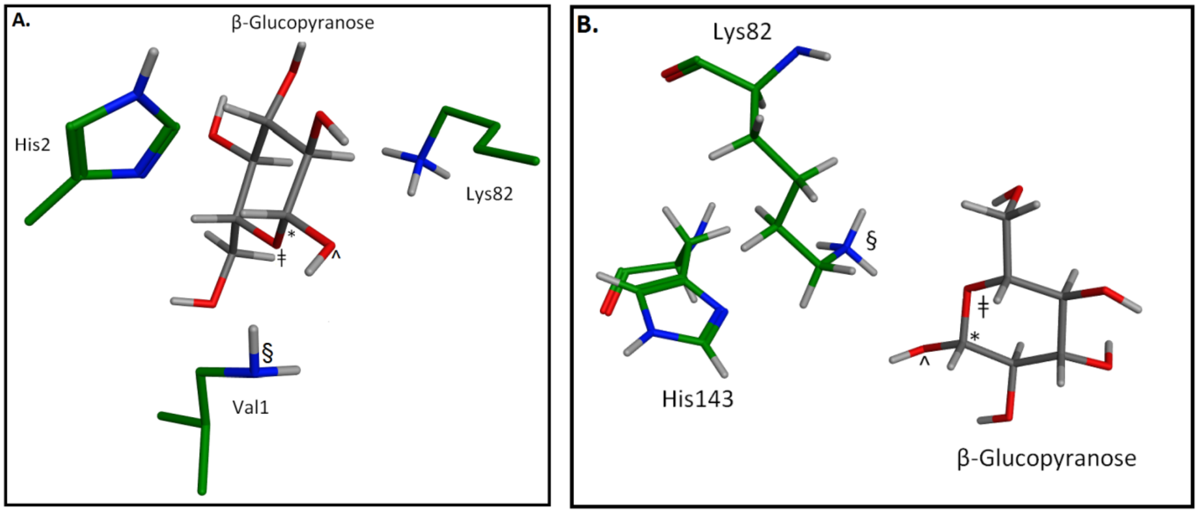

* anomeric carbon; ^ anomeric OH; ‡ hemiacetal oxygen; § nucleophile. Red is oxygen; blue is nitrogen; gray are glucopyranose carbons; green are amino acid residue carbons; white are hydrogens.

In this mechanism, the Lys82 R-NH_3^+ begins to protonate the hemiacetal oxygen of the glucopyranose, 1, (dist. = 3.62Å) while His2 deprotonates the anomeric O<u>H</u> of 1 (dist. = 4.16 Å). These events initiate ring-opening of the glucopyranose, which is then subject to nucleophilic attack at the anomeric carbon by the Val1 R-NH_2 (dist. = 4.12 Å). If the nucleophilic attack is concerted, then the mechanism passes through 2. If the nucleophilic attack is a second step, 2 is generated as an intermediate. Either way, this mechanism leads to a protonated Schiff base.

Lys82 protonates the hemiacetal oxygen (dist. = 2.68Å), His143 deprotonates the anomeric O<u>H</u> (dist. = 4.70Å) generating 2 as an intermediate, and Lys82, now deprotonated, attacks as a nucleophile (dist. = 3.92Å) on route to a Schiff base. This mechanism requires Lys82 to perform a dual-role and thus is likely to be a minor process.

ACCEPTED MANU

Supplementary Figure 4. A 2-dimensional rendering of the singular mechanistic possibility for the formation of HbA1c from 1 in the absence of an effector reagent.

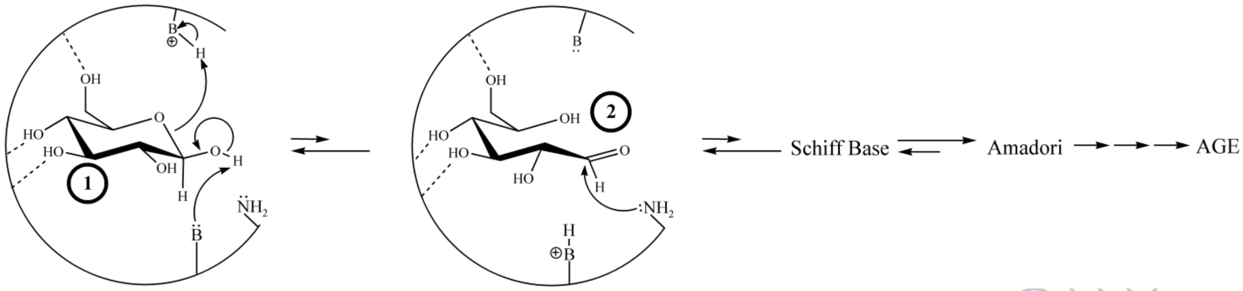

This mechanism is viable for both α- and β-glucopyranose. A His or Lys R-NH_2 amino acid abstracts the anomeric OH of the glucopyranose while the hemiacetal oxygen deprotonates a His-H^+ or Lys R-NH_3^+. This enables a direct nucleophilic attack of the N-terminal Val1 as a nucleophile onto the electrophilic carbon of 2 in the generation of HbA1c. This mechanism corresponds to the MOE geometry illustrated in Supplementary Figure 3a. The probability of 1 binding in the HbA1c pocket with the geometry suitable for this reaction is very low based upon computational assessment. Less than 10% of the computationally determined local minima meet the geometric requirement for this reaction. Therefore, the probability of HbA1c formation from 1 bound alone in the absence of an effector reagent is much lower than in the presence of an effector reagent.

ACCEPTED MANUSCRIPT
